# Cost-Effectiveness of perioperative Vaginally Administered estrogen in postmenopausal women undergoing prolapse surgery (EVA trial): study protocol for a multicenter double-blind randomized placebo-controlled trial

**DOI:** 10.1186/s12905-021-01587-9

**Published:** 2021-12-31

**Authors:** Eva V. Vodegel, Sandra E. Zwolsman, Astrid Vollebregt, Ruben G. Duijnhoven, Judith E. Bosmans, Leonie Speksnijder, Eveline J. Roos, Wilbert Spaans, Franca Gerards, Albert Adriaanse, Flora Vernooij, Alfredo L. Milani, Marko Sikkema, Mirjam Weemhoff, Marieke Mous, Anne Damoiseaux, Heleen van Dongen, Marinus v/d Ploeg, Joggem Veen, Geerte van de Pol, Bart Broekman, Pieternel Steures, Fernando Tjin-Asjoe, Jolande van der Stege, Ronald Mouw, Carl H. van der Vaart, Jan-Paul W. R. Roovers

**Affiliations:** 1 Department of Obstetrics and Gynecology, Amsterdam University Medical Center – Location AMC, University of Amsterdam, Amsterdam, The Netherlands; 2 Department of Obstetrics and Gynecology, Spaarne Gasthuis, Hoofddorp, The Netherlands; 3 Clinical Trials Unit of the Dutch Society for Obstetrics and Gynecology, Amsterdam, The Netherlands; 4 Faculty of Science, Health Economics and Health Technology Assessment, Vrije Universiteit Amsterdam, Amsterdam, The Netherlands; 5 Department of Obstetrics and Gynecology, Amphia Ziekenhuis, Breda, The Netherlands; 6 Department of Obstetrics and Gynecology, Tergooi, Hilversum, The Netherlands; 7 Department of Obstetrics and Gynecology, Maastricht University Medical Center, Maastricht, The Netherlands; 8 Department of Obstetrics and Gynecology, Flevoziekenhuis, Almere, The Netherlands; 9 Department of Obstetrics and Gynecology, Noordwest Ziekenhuisgroep, Alkmaar, The Netherlands; 10 Department of Obstetrics and Gynecology, Diakonessenhuis, Utrecht, The Netherlands; 11 Department of Obstetrics and Gynecology, Reinier de Graaf Gasthuis, Delft, The Netherlands; 12 Department of Obstetrics and Gynecology, Ziekenhuisgroep Twente, Almelo, The Netherlands; 13 Department of Obstetrics and Gynecology, Zuyderland Medisch Centrum, Heerlen, The Netherlands; 14 Department of Obstetrics and Gynecology, Alrijne Ziekenhuis, Leiderdorp, The Netherlands; 15 Department of Obstetrics and Gynecology, Catharina Ziekenhuis, Eindhoven, The Netherlands; 16 Department of Obstetrics and Gynecology, Groene Hart Ziekenhuis, Gouda, The Netherlands; 17 Department of Obstetrics and Gynecology, Martini Ziekenhuis, Groningen, The Netherlands; 18 Department of Obstetrics and Gynecology, Maxima Medisch Centrum, Eindhoven, The Netherlands; 19 Department of Obstetrics and Gynecology, Gelre Ziekenhuis, Apeldoorn, The Netherlands; 20 Department of Obstetrics and Gynecology, Franciscus Gasthuis & Vlietland, Rotterdam, The Netherlands; 21 Department of Obstetrics and Gynecology, Jeroen Bosch Ziekenhuis, ’s Hertogenbosch, The Netherlands; 22 Department of Obstetrics and Gynecology, Maasstad Ziekenhuis, Rotterdam, The Netherlands; 23 Department of Obstetrics and Gynecology, Meander Medisch Centrum, Amersfoort, The Netherlands; 24 Department of Obstetrics and Gynecology, Rijnstate, Arnhem, The Netherlands; 25 Department of Obstetrics and Gynecology, University Medical Center Utrecht, Utrecht, The Netherlands; 26 Bergman Clinics - Vrouw, Amsterdam, The Netherlands

**Keywords:** Pelvic organ prolapse, Vaginal estrogen therapy, Postmenopausal, Wound healing, Pelvic reconstructive surgery, Recurrence, Cost-effectiveness

## Abstract

**Background:**

Surgery for pelvic organ prolapse (POP) is associated with high recurrence rates. The costs associated with the treatment of recurrent POP are huge, and the burden from women who encounter recurrent POP, negatively impacts their quality of life. Estrogen therapy might improve surgical outcome for POP due to its potential beneficial effects. It is thought that vaginal estrogen therapy improves healing and long-term maintenance of connective tissue integrity. Hence, this study aims to evaluate the cost-effectiveness of perioperative vaginal estrogen therapy in postmenopausal women undergoing POP surgery.

**Methods:**

The EVA trial is a multi-center double-blind randomized placebo-controlled trial conducted in the Netherlands comparing the effectiveness and costs-effectiveness of vaginal estrogen therapy. This will be studied in 300 postmenopausal women undergoing primary POP surgery, with a POP-Q stage of ≥ 2. After randomization, participants administer vaginal estrogen cream or placebo cream from 4 to 6 weeks preoperative until 12 months postoperative. The primary outcome is subjective improvement of POP symptoms at 1 year follow-up, measured with the Patient Global Impression of Improvement (PGI-I) scale. Secondary outcomes are POP-Q anatomy in all compartments, re-interventions, surgery related complications, general and disease specific quality of life, sexual function, signs and complaints of vaginal atrophy, vaginal pH, adverse events, costs, and adherence to treatment. Follow up is scheduled at 6 weeks, 6 months and 12 months postoperative. Data will be collected using validated questionnaires and out-patient visits including gynecological examination performed by an independent gynecologist.

**Discussion:**

This study investigates whether perioperative vaginal estrogen will be cost-effective in the surgical treatment of POP in postmenopausal women. It is hypothesized that estrogen therapy will show a reduction in recurrent POP symptoms and a reduction in reoperations for POP, with subsequent improved quality of life among women and cost savings.

*Trial registration*Netherlands Trial Registry: NL6853; registered 19-02-2018, https://www.trialregister.nl/trial/6853. EudraCT: 2017-003144-21; registered: 24-07-2017.

**Supplementary Information:**

The online version contains supplementary material available at 10.1186/s12905-021-01587-9.

## Background

Pelvic organ prolapse (POP) is a common disorder, where 38–50% of postmenopausal women are affected with Stage II prolapse or more [1–4]. Women with POP can experience a sensation of heaviness or bulging in the vagina, and can have problems with voiding, defecation and sexual functioning. These POP-related symptoms adversely affect women’s quality of life, body image as well as their productivity [5–7].

Surgery is often indicated to relieve symptoms, and women have a 11–19% lifetime risk of undergoing surgery for POP [8–10]. However, surgery for POP is associated with high recurrence rates around 30% [9, 11–14], which necessitates additional research to improve surgical outcome for POP.

It is hypothesized that vaginal estrogen therapy might improve surgical outcome for POP and subsequent POP-related symptoms. Yet estrogen therapy has shown to be beneficial for women with divers pelvic floor pathology. Vaginal atrophy can be solved by the use of vaginal estrogen therapy, effectively treating bothersome symptoms like vaginal dryness, and itching of the vulva [15]. In addition, a reduction of symptoms has also been described in women with prolapse symptoms, stress urinary incontinence, overactive bladder symptoms and recurrent cystitis, after using vaginal estrogen [16–18]. The hypothesis is that estrogen results in a thickening of the vaginal wall and urothelium, and improves vascularization of the pelvic floor [18]. Multiple in-depth studies have been performed to investigate the potential beneficial effects of vaginal estrogen therapy [19–21]. A randomized trial showed that the use of vaginal estrogen prior to prolapse surgery increased the production of collagen and reduced degradative enzyme activity [22]. Moreover, estrogens act on the cutaneous wound healing response by modulating the inflammatory response, cytokine expression and matrix deposition [18]. In addition, estrogens accelerate re-epithelialization, stimulating angiogenesis and wound contraction, and regulate proteolysis [18, 23]. Consequently, it is thought that women with low estrogen levels who undergo surgery for POP might have a higher risk of recurrence of their POP—and subsequent symptoms—due to poor vascularization and a thin vaginal wall, compromising their healing capacity. Vaginal estrogen administration is expected to improve healing and long-term maintenance of connective tissue integrity of the pelvic floor [23, 24] and therefore reduce risk of recurrence. Nevertheless, limited supportive data can be found regarding the use of perioperative estrogen to improve clinical outcomes following surgical intervention for prolapse. This was confirmed in a Cochrane review which concluded that a randomized controlled trial is needed to assess perioperative estrogen therapy as adjunctive treatment for women undergoing prolapse surgery [25, 26]. Hence, this study is conducted and aims to evaluate the cost-effectiveness of perioperative vaginal estrogen therapy in postmenopausal women undergoing POP surgery. The results of this study will provide evidence whether the use of estrogen is effective in reducing recurrent POP symptoms, reoperations for POP and hence its cost-effectiveness.

## Methods

### Study design and setting

The EVA trial is a multicenter, double-blind randomized placebo controlled clinical trial. Postmenopausal women undergoing primary POP surgery with a POP-Q stage of ≥ 2, are eligible for the study and will be asked for informed consent to participate. After enrollment, participants are randomized and administer either vaginal estriol or placebo from 4 to 6 weeks before POP surgery till 12 months postoperative.

**Fig. 1:**
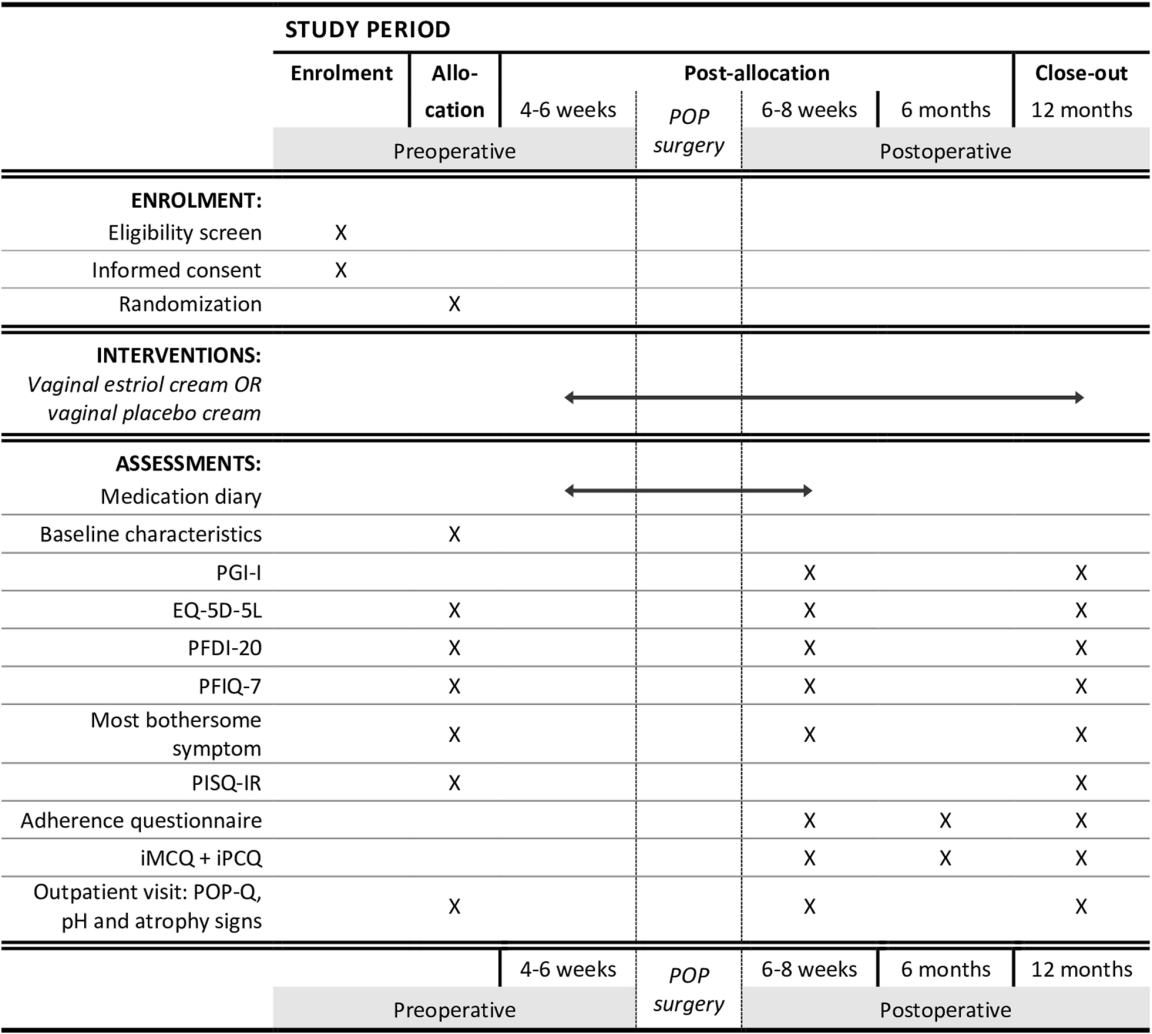
Study design: schedule of enrolment, interventions and assessments. *EQ-5D-5L* emotional quotient, 5 dimensions, 5 levels, *iMCQ* Medical Consumption Questionnaire, *iPCQ* Productivity Cost Questionnaire, *PFDI-20* Pelvic Floor Disability Index-20, *PFIQ-7* Pelvic Floor Impact Questionnaire-7, *PGI-I* Patient Global Impression of Improvement, *PISQ-IR* Pelvic organ prolapse Incontinence Sexual Questionnaire-IR, *POP* Pelvic Organ Prolapse, *POP-Q* Pelvic Organ Prolapse Quantification

Recruitment will take place in a multicenter setting in 22 participating centers in the Netherlands, i.e. university, teaching and non-teaching hospitals. A list of current study sites can be obtained via the website [27]. Gynecologists and residents, supported by research nurses, will counsel women, ask informed consent, perform randomization and collect data. Both participants and investigators will be blinded for allocation. The study design is presented in Fig. 1. The study is conducted in cooperation with the urogynecology consortium of the Netherlands and the Dutch NVOG Consortium 2.0 (Dutch Consortium for Healthcare Evaluation and Research in Obstetrics and Gynecology).

### Participants and eligibility criteria

#### Inclusion criteria

- Postmenopausal women (> 1 year amenorrhea)
- Pelvic organ prolapse; POP-Q stage ≥ 2 [28]
- Women that will undergo primary POP surgery with native tissue repair, including at least anterior OR posterior vaginal wall repair

#### Exclusion criteria

- Previous POP surgery in concerning compartment;
- Prolapse repair using mesh
- Current vaginal infection
- Use of estrogens in the past 12 months
- Contraindications for topical estrogen
- Known, past or suspected estrogen-dependent malignant tumors
- Insufficient knowledge or understanding of the Dutch language

### Interventions

After obtaining informed consent, eligible women will be assigned to either estriol cream (Synapause-E3; 1 mg/g) or placebo cream. Both creams are vaginally administered, ante noctem and come with a special applicator. The applicator allows the participant to insert the correct amount of cream deep into the vagina. Therefore, the participant lies on her back with slightly bent, spread legs. The cream-filled applicator is applied deep into the vagina. Then the applicator will be emptied and removed from the vagina. During the first 2 weeks of use, 0.5 g cream will be used once a day. Thereafter, 0.5 g cream will be used vaginally twice per week. The first 2 weeks postoperative, no cream will be used. The total duration of this intervention will be 58 weeks; 6 weeks prior to POP surgery (with a minimum of 4 weeks) and 52 weeks after POP surgery. This dosage schedule corresponds with the standard dosage schedule used in clinical practice. Women will receive routine clinical care and remain blinded to treatment allocation until the primary analysis has been completed.

Preparation and labelling of study medication will be done according to Good Manufacturing Practice guidelines. Manufacturing and packaging of both estriol vaginal cream (1 mg/g) and placebo cream is performed by Aspen Pharmacare. During the manufacturing process of estriol vaginal cream and placebo cream, the exact same ingredients were used, except from estriol. The active product ingredient estriol is no component in the placebo cream. Labelling is performed by the pharmacy of the Academic Medical Center Amsterdam.

#### Low-dose vaginal estrogen

The low-dose vaginal estrogen used in this study is preferred over systemic (oral) estrogens, because systemic use is associated with an increased risk of thrombosis and endometrial, breast and ovarian cancer. Low-dose regimens are also preferred over intermediate- or high dose methods since absorption and systemic effects occur with high-dose vaginal estrogen preparations [29–34]. Although vaginally administered low-dose estrogen could result in a minimal systemic uptake, serum estrogen levels remain within the normal range for postmenopausal women and no endometrial or myometrial effects are seen [22, 35–40]. Therefore, vaginal estrogens are an effective and safe strategy and is in line with the global consensus statement on menopausal hormonal therapy, where low-dose estrogen is preferred [41].

### Outcome measures

The primary outcome is subjective improvement of POP symptoms at 1 year follow-up; the percentage of women with much or very much improvement of POP symptoms, measured with the Patient Global Impression of Improvement (PGI-I) scale [42]. PGI-I is a 7-point Likert scale, with scores ranging from very much worse to very much improved. Success is defined as ‘much or very much’ improvement.

Secondary outcomes are surgical success, POP-Q anatomy in all compartments, surgery related complications, general quality of life, disease specific quality of life (micturition and defecation), sexual function, signs and complaints of vaginal atrophy, vaginal pH, adverse events, costs, and adherence to treatment. Surgical success is defined as the absence of POP beyond the hymen (POP-Q at gynecological examination), the absence of bulge symptoms (absence of bulge symptoms is defined as a negative response to the question, “Do you see or feel a bulge in the vaginal area”), and absence of reoperation or additional pessary therapy for POP [43]. Follow up is scheduled at 6 weeks, 6 months and 12 months postoperative. Data will be collected using validated questionnaires (See also ‘Data collection’ and Fig. 1) and out-patient visits including gynecological examination are performed by an independent gynecologist.

### Patient recruitment and consent

Eligible women receive oral and written information on study participation during a regular hospital visit for POP complaints. Women will be contacted by telephone for further information by the gynecologist, resident or research nurse. Women will be given sufficient time to read the patient information and the informed consent form and get the opportunity to ask questions. If a woman agrees to participate in the study, written informed consent will be obtained and countersigned by the investigator where after randomization will be performed, according to Good Clinical Practice (GCP).

### Randomization

Women will be randomized in a 1:1 ratio to perioperative treatment with estriol or perioperative treatment with placebo using computer-generated randomly permuted blocks of sizes 4, 6 and 8. Blinded randomization will be performed by the participating center using online software (Castor Electronic Data Capture, Amsterdam, the Netherlands [online] available at: http://castoredc.com). The central coordinating pharmacy of the Academic Medical Center Amsterdam receives an automatically generated email containing the allocated treatment for each randomized patient from the Castor system. After also receiving a doctor’s prescription, the pharmacy will deliver study medication at the participant’s home address by courier service.

### Blinding

Study medication will be blinded. Both estriol cream and placebo cream are provided to the participant in identical blank white tubes, marked with a study label. All physicians, researchers, research nurses, outcome assessors and participants will remain blinded to treatment allocation until the primary analysis has been completed. During the conduct of the study, only the pharmacy of the Academic Medical Center Amsterdam will have access to the blinding information of the study. With the approval of the principal investigator, a local investigator could decide to reveal a participant’s allocation for urgent medical reasons only. Unblinding will be performed by the central coordinating pharmacy at the Academic Medical Center Amsterdam.

### Data collection

All data will be systematically recorded by trained research nurses or gynecologists in an electronic Case Report Form in web-based data management software: Castor EDC. Castor EDC allows researchers to collect and manage data in accordance with the GCP guidelines [44]. To improve data quality, range checks are incorporated in the electronic Case Report Form. The software will randomly assign an unique numeric code for every subject that bears no relation to initials or date of birth. Data handling will be done with coded data, with the key (code to personal information linkage) only available to the local investigator and the research nurse working in the participating center. Persons who have access to the coded data include: investigators, research staff, monitoring and quality assurance personal. Data will be preserved for the duration of 15 years. The handling of personal data complies with the European General Data Protection Regulation. Participants will be followed from baseline (pre-operatively) up to 1 year after POP surgery. During the follow up period the following data will be collected:

#### Hospital visits

The participant will visit the hospital prior to starting with the study medication, 6 weeks postoperative and 12 months postoperative. History and gynecological examination including POP-Q, vaginal pH and assessment of vaginal atrophy will be performed. To reduce interviewer bias, the POP-Q measurement at 12 months postoperative will be done by an independent examiner—instead of the surgeon. Unscheduled visits and phone calls are also recorded in respect to additional costs.

#### Surgery and postoperative data

Surgery time, blood loss, surgery related complications (including excessive blood loss, hematoma, lesion of the gut, bladder, ureter or urethra, urinary retention, urinary tract infection, and prolonged hospitalization) are recorded.

#### Questionnaires and diary

Participants will be asked to complete online questionnaires, generated in Castor EDC (when preferred, it is possible to complete the questionnaires on paper). Participants will receive 2 or more of the following questionnaires at baseline and 6 weeks, 6 months and 12 months postoperative (See also Fig. 1):

- Questionnaire on baseline characteristics and medical history (including age, length, weight, parity, education, history of diabetes, respiratory diseases and smoking, family history of POP).
- Patient Global Impression of Improvement (PGI-I): to assess subjective improvement of POP symptoms [42].
- EuroQoL (EQ-5D-5L): a general quality of life questionnaire, to evaluate health utilities and the corresponding quality adjusted life years (QALYs) [45].
- Pelvic Floor Disability Index-20 (PFDI-20): to measure symptom distress on disease specific quality of life, focusing on POP, urinary incontinence, and fecal incontinence [46].
- Pelvic Floor Impact Questionnaire-7 (PFIQ-7): to measure impact on specific quality of life, focusing on POP, urinary incontinence, and fecal incontinence [46].
- Most Bothersome Symptom questionnaire: to assess the most bothersome urogenital symptoms [47].
- The Pelvic organ prolapse Incontinence Sexual Questionnaire-IR (PISQ-IR): a condition-specific measure of sexual function in women with pelvic floor dysfunction, including urinary and anal incontinence and pelvic organ prolapse [48].
- Medical Consumption Questionnaire (iMCQ): for cost effectiveness analysis [49].
- Productivity Cost Questionnaire (iPCQ) to assess productivity loss [50].
- Adherence questionnaire: on adherence to treatment, experience using perioperative vaginal cream and influencing factors on compliance. This questionnaire has been developed for this study and is provided as Additional file 1.
- Adherence diary: a perioperative diary will be kept on the use of vaginal cream, from start till 6 weeks postoperative. Adverse reactions associated with vaginally administered estriol cream (e.g. temporary itch or irritation of the application site) can be reported in the diary as well.
- Adherence to treatment: Study medication will be delivered at the participant’s home address at two time points; 2 tubes at randomization and 3 tubes at 5 months post-randomization. Participants are asked to return the study medication at 5 months post-randomization and at the end of the study—12 months post-operative. In order to asses adherence to treatment, tubes will be weighted on a scale (Denver Instrument Company, TR-602, digit: 0.01 g) and recorded in Castor EDC.

In case of non-completion of questionnaires, a first reminder will be sent by email after two weeks. If necessary, a second reminder will be sent after 3 weeks. In case of persistent non-completion after 4 weeks, the participant is contacted by telephone by the research nurse.

#### Long-term outcomes

The intention is to evaluate the long-term outcomes of the participants as well (e.g. 3 or 5 years postoperative). When this seems valuable after the 1 year analysis, MEC approval will be obtained in a separate amendment. Permission to approach the participants for this follow-up research is obtained at the time of the informed consent procedure.

### Monitoring and safety

To assure high quality research data and secure patient safety, adequate monitoring will be performed in accordance with the GCP guidelines. Monitoring will be coordinated by the Dutch NVOG Consortium 2.0 and will be executed by a qualified independent monitor. Based on the NVOG Site Monitoring Plan 3.1 of the Dutch NVOG Consortium, remote initiation visits and monitoring visits in each participating center will be performed every year. The independent monitor will have access to the data and source documents of the trial to review the quality of the participating centers. More detailed information can be found in the monitoring plan of the study via www.zorgevaluatienederland.nl/eva. Serious adverse events and any other significant problems will be reported to the medical ethics committee of the Amsterdam Medical Center. A data safety monitoring board will not be installed for this study since perioperative topical estrogens are commonly used in daily practice and use within this study is not perceived to pose an additional risk. Also no interim analysis for efficacy will be performed.

### Insurance

According to national guidelines, a liability insurance and also an insurance that covers injury to participants caused by this study are taken out. These insurances cover injuries up to 4 years after the end of the study.

### Statistical analysis

#### Sample size

The sample size is based on the primary endpoint of subjective cure rate (PGI-I) at 12 months postoperative. A difference in subjective cure of more than 15% is considered clinically relevant. Assuming a subjective cure of 80% in the intervention group and a subjective cure of 65% in the control group; 136 women in each trial arm are needed to assess superiority of the intervention, with a power of 80% and an α of 0.05. With an expected 10% loss to follow up, a number of 150 women will be included in each trial arm (total sample size 300 women).

#### Data analysis

Both an intention-to-treat analysis and per protocol analysis will be performed to evaluate the effect of perioperative estrogen; taking possible confounders in consideration such as study withdrawal and missing data. Analyses will be done using SPSS version 26.0. A *p* value of < 0.05 is considered a threshold for significance. Baseline characteristics will be presented using descriptive statistics, with frequencies (numbers, mean or median with respectively percentages, standard deviation or quartiles).

##### Primary study parameter

The primary outcome, subjective cure (PGI-I), will be reported as frequency with percentage and relative risk with 95% confidence interval together with a *p* value for a chi-squared test. Patients are considered to be cured when they state to be “much better” and “very much better” on a 7 point Likert scale.

##### Secondary study parameters

All the secondary outcomes will be presented as frequencies with percentages for dichotomous outcomes, means with standard deviations for continuous normally distributed variables and medians with interquartile ranges for non-normally distributed variables.

We plan to calculate the differences within groups between baseline and 12 months using either a paired t-test, Wilcoxon signed rank test or McNemar test for PFIQ, PFDI-20, PISQ-IR, the compound measure, vaginal pH, vaginal atrophy (objective and subjective outcomes), adverse events, gynecological interventions performed, costs and compliance. Differences between groups (including relative risks with 95% confidence interval) will be analyzed using one-sample t-test for normally distributed numerical data, Mann–Whitney U tests for not normally distributed numerical data, chi-square testing for categorical data and Fisher’s exact testing for rare outcomes.

The secondary outcome quality of life at 1 year after the procedure, will be measured with the EQ-5D-5L and will be analyzed using the utility calculation that was used in other studies [51]. Quality of life outcomes will be described and analyzed accordingly. Difference in utilities between groups will be estimated using a linear regression. For each time point, QALYs will be calculated by multiplying the utilities by time (as fraction of a year) [52, 53]. Results will be further incorporated in the cost-effectiveness analyses.

Adherence to therapy will be assessed by comparing tube weights to the anticipated weight if used according to protocol. Participants will also keep a perioperative diary on the use of vaginal cream to calculate adherence to therapy until 6 weeks postoperative. Moreover, adherence to therapy will be calculated at 6 and 12 months postoperative from the adherence questionnaire.

### Economic evaluation

A cost-effectiveness analysis (CEA) will be performed based on empirical data obtained in the study. The CEA will take a societal and health care perspective, involving direct medical (health care related), non-medical (travel, over the counter medication, time costs), and indirect (productivity) costs. The primary outcome measure in the CEA will be the PFDI-20, whereas cost-effectiveness will be expressed as costs per unit increase on the PFDI-20-scale. As most anatomical failures occur in the first year after surgery, we will assess the primary endpoint at 12 months after surgery. In line with the clinical endpoints, secondary outcome measures will be satisfaction with treatment and QALY, with cost-effectiveness ratios expressed as costs per woman satisfied with treatment and costs per QALY. The clinical outcomes will be derived from the trial data. Health state utilities to estimate QALYs will be derived from EQ-5D measurements at baseline, as well as 12 months. Utility values for EQ-5D scores will be based on Dutch estimates. The cost-utility analysis will estimate Incremental costs per QALYs will be calculated to determine cost-utility [54]. Costs will be calculated by multiplying resource use per patient with unit-cost estimates. Resource use will be obtained from trial records, complemented with patient administered cost-questionnaires. Unit costs will be based on publicly available standardized costs, fee schedules, and estimates reported in the medical literature. The choice of costing method will depend on the availability of appropriate cost estimates. Incremental costs and effects will be illustrated in a scatter plot to shown cost-effectiveness of the interventon as compared to control [54]. Neither costs nor effects will be discounted as the time horizon of the analyses is limited to 1 year. Sensitivity analyses will be performed to assess robustness of the results for uncertainties and assumptions regarding unit costs and resource use: bootstrap analysis (1000 replicates) will be used to estimate uncertainty [54]. Long term outcomes (for both costs and clinical outcomes) will be evaluated using modelling techniques. Consequences for the Dutch health care budget will be estimated in a budget impact analysis.

## Dissemination plan

Dissemination of the study results will be obtained by publication in an international peer-reviewed scientific journal and by presentations at (inter)national conferences. Second, the results will be translated into clear recommendations for the national clinical guideline. Third, the results will be distributed among participants and incorporated in education programs. Finally, experience shows that involvement in evidence collection has a positive effect on the implementation of the results [55]. Consequently, in our efforts towards generalizability and the implementability of the results, we aim to conduct this study in a multi-center setting where all participating centers are member of the NVOG Consortium 2.0. Currently, 22 centers in the Netherlands are participating, making this project a major national multi-center trial. Further on, a patient preference evaluation will be performed within this study, where participants are asked about their experiences using perioperative vaginal cream, to what extend the use of vaginal cream weights against the outcome of the surgery and influencing factors on compliance. This patient preference evaluation helps to identify potential barriers which will facilitate and optimize further implementation.

## Discussion

The results of this study will provide evidence whether perioperative vaginal estrogen therapy will be effective in reducing recurrent POP symptoms and reoperations for POP with subsequent improved quality of life among women and cost savings; a simple, cheap and safe intervention with potential impact in clinical practice. Since there is no consensus regarding the clinical benefits of perioperative vaginal estrogen therapy in postmenopausal women with prolapse, the current Dutch clinical guideline on POP does not include a recommendation on the use of estrogen before and after prolapse surgery. This results in a high variability in current practice based on doctors’ preference mainly. Estrogens are prescribed preoperatively, postoperatively, perioperatively or not at all. For that reason, the results of this study could be used to formulate a uniform policy regarding vaginal estrogen therapy for primary POP surgery in the Netherlands, and possibly on international level as well.

## Supplementary Information

**Additional file 1**. Title: Adherence questionnaire (translated to English). Description: Questionnaire (developed for this study) on adherence to treatment, experience using perioperative vaginal cream and influencing factors on compliance.

## Abbreviations

Castor EDC: Castor electronic data capture
CEA: Cost effectiveness analysis
EQ-5D-5L: Emotional Quotient, 5 Dimensions, 5 Levels
GCP: Good Clinical Practice
iMCQ: Medical Consumption Questionnaire
iPCQ: Productivity Cost Questionnaire
MEC: Medical Review Ethics Committee
NVOG Consortium: Nederlandse Vereniging voor Obstetrie en Gynaecologie Consortium
PFDI-20: Pelvic Floor Disability Index-20
PFIQ-7: Pelvic Floor Impact Questionnaire-7
PGI-I: Patient Global Impression of Improvement
PISQ-IR: Pelvic organ prolapse Incontinence Sexual Questionnaire-IR
POP: Pelvic Organ Prolapse
POP-Q: Pelvic Organ Prolapse Quantification
QALY: Quality Adjusted Life Years
RCT: Randomized controlled trial

## Declarations

### Availability of data and materials

The datasets used and/or analyzed during the current study will be available from the corresponding author on reasonable request.

## References

[1] Slieker-ten Hove MC, Pool-Goudzwaard AL, Eijkemans MJ, Steegers-Theunissen RP, Burger CW, Vierhout ME (2009). The prevalence of pelvic organ prolapse symptoms and signs and their relation with bladder and bowel disorders in a general female population. Int Urogynecol J Pelvic Floor Dysfunct.

[2] Hendrix SL, Clark A, Nygaard I, Aragaki A, Barnabei V, McTiernan A (2002). Pelvic organ prolapse in the Women's Health Initiative: gravity and gravidity. Am J Obstet Gynecol.

[3] Swift S, Woodman P, O'Boyle A, Kahn M, Valley M, Bland D (2005). Pelvic Organ Support Study (POSST): the distribution, clinical definition, and epidemiologic condition of pelvic organ support defects. Am J Obstet Gynecol.

[4] Swift SE (2000). The distribution of pelvic organ support in a population of female subjects seen for routine gynecologic health care. Am J Obstet Gynecol.

[5] Committee on Practice Bulletins-Gynecology, American Urogynecologic Society (2017). Practice bulletin no. 185: pelvic organ prolapse. Obstet Gynecol.

[6] Zielinski R, Low LK, Tumbarello J, Miller JM (2009). Body image and sexuality in women with pelvic organ prolapse. Urol Nurs.

[7] Tso C, Lee W, Austin-Ketch T, Winkler H, Zitkus B (2018). Nonsurgical treatment options for women with pelvic organ prolapse. Nurs Womens Health.

[8] Wu JM, Matthews CA, Conover MM, Pate V, Jonsson FM (2014). Lifetime risk of stress urinary incontinence or pelvic organ prolapse surgery. Obstet Gynecol.

[9] Olsen AL, Smith VJ, Bergstrom JO, Colling JC, Clark AL (1997). Epidemiology of surgically managed pelvic organ prolapse and urinary incontinence. Obstet Gynecol.

[10] Smith FJ, Holman CD, Moorin RE, Tsokos N (2010). Lifetime risk of undergoing surgery for pelvic organ prolapse. Obstet Gynecol.

[11] Nygaard I, Brubaker L, Zyczynski HM, Cundiff G, Richter H, Gantz M (2013). Long-term outcomes following abdominal sacrocolpopexy for pelvic organ prolapse. JAMA.

[12] Wong V, Shek KL, Goh J, Krause H, Martin A, Dietz HP (2014). Cystocele recurrence after anterior colporrhaphy with and without mesh use. Eur J Obstet Gynecol Reprod Biol.

[13] Whiteside JL, Weber AM, Meyn LA, Walters MD (2004). Risk factors for prolapse recurrence after vaginal repair. Am J Obstet Gynecol.

[14] Aslam MF, Osmundsen B, Edwards SR, Matthews C, Gregory WT (2016). Preoperative prolapse stage as predictor of failure of sacrocolpopexy. Female Pelvic Med Reconstr Surg.

[15] Lethaby A, Ayeleke RO, Roberts H (2016). Local oestrogen for vaginal atrophy in postmenopausal women. Cochrane Database Syst Rev.

[16] Weber MA, Kleijn MH, Langendam M, Limpens J, Heineman MJ, Roovers JP (2015). Local oestrogen for pelvic floor disorders: a systematic review. PLoS One..

[17] Cody JD, Jacobs ML, Richardson K, Moehrer B, Hextall A (2012). Oestrogen therapy for urinary incontinence in post-menopausal women. Cochrane Database Syst Rev.

[18] Krause M, Wheeler TL, Snyder TE, Richter HE (2009). local effects of vaginally administered estrogen therapy: a review. J Pelvic Med Surg.

[19] Moalli PA, Shand SH, Zyczynski HM, Gordy SC, Meyn LA (2005). Remodeling of vaginal connective tissue in patients with prolapse. Obstet Gynecol.

[20] Kerkhof MH, Ruiz-Zapata AM, Bril H, Bleeker MC, Belien JA, Stoop R (2014). Changes in tissue composition of the vaginal wall of premenopausal women with prolapse. Am J Obstet Gynecol.

[21] De Landsheere L, Munaut C, Nusgens B, Maillard C, Rubod C, Nisolle M (2013). Histology of the vaginal wall in women with pelvic organ prolapse: a literature review. Int Urogynecol J.

[22] Rahn DD, Good MM, Roshanravan SM, Shi H, Schaffer JI, Singh RJ (2014). Effects of preoperative local estrogen in postmenopausal women with prolapse: a randomized trial. J Clin Endocrinol Metab.

[23] Ashcroft GS, Ashworth JJ (2003). Potential role of estrogens in wound healing. Am J Clin Dermatol.

[24] Mukai K, Urai T, Asano K, Nakajima Y, Nakatani T (2016). Evaluation of effects of topical estradiol benzoate application on cutaneous wound healing in ovariectomized female mice. PLoS One..

[25] Ismail SI, Bain C, Hagen S. Oestrogens for treatment or prevention of pelvic organ prolapse in postmenopausal women. Cochrane Database Syst Rev. 2010;9:CD007063. 10.1002/14651858.CD007063.pub2 PMC13590214 20824855

[26] Karp DR, Jean-Michel M, Johnston Y, Suciu G, Aguilar VC, Davila GW (2012). A randomized clinical trial of the impact of local estrogen on postoperative tissue quality after vaginal reconstructive surgery. Female Pelvic Med Reconstr Surg.

[27] Dutch Society of Obstetrics and Gynaecology's (NVOG) clinical trial consortium. https://zorgevaluatienederland.nl/evaluations/eva. Accessed 1 Oct 2021.

[28] Bump RC, Mattiasson A, Bo K, Brubaker LP, DeLancey JO, Klarskov P (1996). The standardization of terminology of female pelvic organ prolapse and pelvic floor dysfunction. Am J Obstet Gynecol.

[29] Suckling J, Lethaby A, Kennedy R. Local oestrogen for vaginal atrophy in postmenopausal women. Cochrane Database Syst Rev. 2006;4:CD001500. 10.1002/14651858.CD001500.pub2 17054136

[30] Krause M, Wheeler TL, Richter HE, Snyder TE (2010). Systemic effects of vaginally administered estrogen therapy: a review. Female Pelvic Med Reconstr Surg.

[31] Al-Azzawi F, Lees B, Thompson J, Stevenson JC (2005). Bone mineral density in postmenopausal women treated with a vaginal ring delivering systemic doses of estradiol acetate. Menopause.

[32] Al-Azzawi F, Buckler HM (2003). Comparison of a novel vaginal ring delivering estradiol acetate versus oral estradiol for relief of vasomotor menopausal symptoms. Climacteric.

[33] Crandall C (2002). Vaginal estrogen preparations: a review of safety and efficacy for vaginal atrophy. J Women's Health (2002).

[34] (2013) Management of symptomatic vulvovaginal atrophy: 2013 position statement of The North American Menopause Society. Menopause 20(9):888–902 **(quiz 3–4**) 10.1097/GME.0b013e3182a122c2 23985562

[35] Lupo M, Dains JE, Madsen LT (2015). Hormone replacement therapy: an increased risk of recurrence and mortality for breast cancer patients?. J Adv Pract Oncol.

[36] Farrell R (2016). ACOG Committee Opinion No. 659 Summary: the use of vaginal estrogen in women with a history of estrogen-dependent breast cancer. Obstet Gynecol.

[37] Le Ray I, Dell'Aniello S, Bonnetain F, Azoulay L, Suissa S (2012). Local estrogen therapy and risk of breast cancer recurrence among hormone-treated patients: a nested case-control study. Breast Cancer Res Treat.

[38] Mazzarello S, Hutton B, Ibrahim MFK, Jacobs C, Shorr R, Smith S (2015). Management of urogenital atrophy in breast cancer patients: a systematic review of available evidence from randomized trials. Breast Cancer Res Treat.

[39] Dew JE, Wren BG, Eden JA (2003). A cohort study of topical vaginal estrogen therapy in women previously treated for breast cancer. Climacteric.

[40] Santen RJ (2015). Vaginal administration of estradiol: effects of dose, preparation and timing on plasma estradiol levels. Climacteric.

[41] de Villiers TJ, Gass ML, Haines CJ, Hall JE, Lobo RA, Pierroz DD (2013). Global consensus statement on menopausal hormone therapy. Climacteric.

[42] Srikrishna S, Robinson D, Cardozo L (2010). Validation of the patient global impression of improvement (PGI-I) for urogenital prolapse. Int Urogynecol J.

[43] Barber MD, Brubaker L, Nygaard I, Wheeler TL, Schaffer J, Chen Z (2009). Defining success after surgery for pelvic organ prolapse. Obstet Gynecol.

[44] https://www.castoredc.com/blog/how-do-you-perform-gcp-compliant-research-with-castor-edc/

[45] Janssen MF, Pickard AS, Golicki D, Gudex C, Niewada M, Scalone L (2013). Measurement properties of the EQ-5D-5L compared to the EQ-5D-3L across eight patient groups: a multi-country study. Qual Life Res.

[46] Utomo E, Blok BF, Steensma AB, Korfage IJ (2014). Validation of the Pelvic Floor Distress Inventory (PFDI-20) and Pelvic Floor Impact Questionnaire (PFIQ-7) in a Dutch population. Int Urogynecol J.

[47] Simon J, Nachtigall L, Gut R, Lang E, Archer DF, Utian W (2008). Effective treatment of vaginal atrophy with an ultra-low-dose estradiol vaginal tablet. Obstet Gynecol.

[48] van Dongen H, van der Vaart H, Kluivers KB, Elzevier H, Roovers JP, Milani AL (2018). Dutch translation and validation of the pelvic organ prolapse/incontinence sexual questionnaire-IUGA revised (PISQ-IR). Int Urogynecol J.

[49] Bouwmans C, Roijen L, Koopmanschap M, Krol M, Severens H, Brouwer W et al (2013) Handleiding iMTA Medical Cost Questionnaire (iMCQ). Erasmus Universiteit Rotterdam: iMTA. www.imta.nl

[50] Bouwmans C, Krol M, Severens H, Koopmanschap M, Brouwer W, Hakkaart-van RL (2015). The iMTA Productivity Cost Questionnaire: a standardized instrument for measuring and valuing health-related productivity losses. Value Health.

[51] Cost effective self-management of urinary incontinence addressed to women across Europe. Estimated cost-effectiveness of using the proposed care model as a primary treatment, compared to care as usual. Women-up Consortium. 2019

[52] Weatherly H, Drummond M, Claxton K, Cookson R, Ferguson B, Godfrey C (2009). Methods for assessing the cost-effectiveness of public health interventions: key challenges and recommendations. Health Policy.

[53] Whitehead SJ, Ali S (2010). Health outcomes in economic evaluation: the QALY and utilities. Br Med Bull.

[54] Labrie J, van der Graaf Y, Buskens E, Tiersma SE, van der Vaart HC (2009). Protocol for Physiotherapy Or TVT Randomised Efficacy Trial (PORTRET): a multicentre randomised controlled trial to assess the cost-effectiveness of the tension free vaginal tape versus pelvic floor muscle training in women with symptomatic moderate to severe stress urinary incontinence. BMC Womens Health.

[55] Aarts JW, Faber MJ, den Boogert AG, Cohlen BJ, van der Linden PJ, Kremer JA (2013). Barriers and facilitators for the implementation of an online clinical health community in addition to usual fertility care: a cross-sectional study. J Med Internet Res.

