# Supplementary material for: Cost-Effectiveness of perioperative Vaginally Administered estrogen in postmenopausal women undergoing prolapse surgery (EVA trial): study protocol for a multicenter double-blind randomized placebo-controlled trial
Source: BMC Womens Health. 2021 Dec 31;21:439. doi: 10.1186/s12905-021-01587-9 (PMC8720212; doi:10.1186/s12905-021-01587-9)
Supplement: Supplementary file 1 — Additional file 1. Title: Adherence questionnaire (translated to English). Description: Questionnaire (developed for this study) on adherence to treatment, experience using perioperative vaginal cream and influencing factors on compliance. [file 12905_2021_1587_MOESM1_ESM.pdf]

Survey: Adherence to treatment

It can sometimes be difficult to use medication on a regular schedule. We are interested in your experience with the ***vaginal cream*** *in the past 2 months. Try to answer the questions as honestly as possible. There is no right or wrong answer.*

	Never/rarely	Occasionally	Sometimes	Often	(almost) Always
**Question 1. How often did you forget to administer the vaginal cream?** *(in the past 2 months)*	☐	☐	☐	☐	☐

Question 2. Did you administer the vaginal cream according schedule to last week?

- ☐ Yes
- ☐ No

Question 3. Was it difficult for you to stick to the prescribed administration schedule?
(in the past 2 months)

- ☐ Yes
- ☐ No

In the past 2 months, to what extent were the following points a reason for you not to administer the vaginal cream?

		Never	Rarely	Sometimes	Often
**4.**	I forgot	☐	☐	☐	☐
**5.**	No/ insufficient vaginal cream was delivered by the pharmacy	☐	☐	☐	☐
**6.**	I was not at home	☐	☐	☐	☐
**7.**	I had to take several meds a day	☐	☐	☐	☐
**8.**	I wanted to avoid side effects	☐	☐	☐	☐
**9.**	I didn't want other people to see that I'm on medication	☐	☐	☐	☐
**10.**	I was too tired to administer the cream	☐	☐	☐	☐
**11.**	I found the use of the vaginal cream too much effort	☐	☐	☐	☐
**12.**	I didn't feel well (cold, flu, etc.)	☐	☐	☐	☐
**13.**	I felt down / depressed	☐	☐	☐	☐
**14.**	Administering the cream is not part of my routine	☐	☐	☐	☐
**15.**	I feel healthy (so have no reason to use the vaginal cream)	☐	☐	☐	☐

		Never	Rarely	Sometimes	Often
**16.**	I felt that the cream did not had an effect	☐	☐	☐	☐
**17.**	I found the time of administration difficult	☐	☐	☐	☐
**18.**	Otherwise, namely …..	☐	☐	☐	☐

In the past 2 months:	**Never /rarely**	**Occasionally**	**Some times**	**Often**	**(almost) Always**
19. Was applying the vaginal cream uncomfortable or painful?	☐	☐	☐	☐	☐
**20. A)** Did the cream come out of your vagina after insertion?	☐	☐	☐	☐	☐
**B)** If yes, did you find this unpleasant?	*No* ☐	*Somewhat* ☐	*Quite* ☐	*Fair* ☐	*N/A* ☐
**21.** If the cream came out of your vagina, was it such that you needed a tissue/washcloth to clean it? ☐ N/A	☐	☐	☐	☐	☐

Question 22. How easy is the application of the vaginal cream?

☐ Easy
☐ Reasonable
☐ Difficult

Local estrogen therapy is available as a vaginal cream but also as a vaginal tablet. Just like the cream, you insert the tablet deep into the vagina (with your finger or with an applicator). The schedule for both the cream and the tablet is the same.

Question 23. If you could choose a vaginal cream or a vaginal tablet, which would you prefer?

☐ Cream
☐ Tablet

Question 24. Would you be willing to use vaginal estrogen cream or tablets (after your prolapse surgery) for a year (twice a week) if the risk of recurrent pelvic organ prolapse decreases from 20% to 10%?

☐ Yes
☐ No
